# Association between neutrophil-to-lymphocyte ratio, platelet-to-lymphocyte ratio, and diabetic retinopathy among diabetic patients without a related family history

**DOI:** 10.1186/s13098-020-00562-y

**Published:** 2020-07-02

**Authors:** Jin-Rui Wang, Zhongli Chen, Ke Yang, Hui-Jun Yang, Wen-Yu Tao, Yi-Ping Li, Ze-Jia Jiang, Chao-Fang Bai, Yue-Chuan Yin, Jian-Mei Duan, Yuan-Yuan Zhou, Xin-Qian Geng, Ying Yang

**Affiliations:** 1 Department of Endocrinology and Metabolism, The Second People’s Hospital of Yunnan Province, Fourth Affiliated Hospital of Kunming Medical University, Kunming, 650021 China; 2 Department of Cardiology, Ruijin Hospital, Shanghai Jiaotong University School of Medicine, 200001 Shanghai, China; 3 The Second People’s Hospital of Qujing City, Yunnan Qujing, 655000 China

**Keywords:** Type 2 diabetes mellitus, Diabetic retinopathy, Family history, Neutrophil-to-lymphocyte ratio, Platelet-to-lymphocyte ratio, Hemoglobin

## Abstract

**Background:**

Diabetic retinopathy (DR) is a specific neurovascular complication of diabetes mellitus (DM). Clinically, family history is a widely recognized risk factor for DR, assisting diagnosis and risk strata. However, among a great amount of DR patients without hereditary history like hypertension and diabetes, direct and simple risk factors to assist clinical decisions are still required. Herein, we intend to investigate the associated risk factors for these DR patients based on systemic inflammatory response indexes, neutrophil-to-lymphocyte ratio (NLR) and platelet-to-lymphocyte ratio (PLR).

**Methods:**

We consecutively enrolled 1030 patients with a definite diagnosis of type 2 diabetes mellitus (T2DM) from the endocrinology department of the Second hospital of People in Yun Nan. Based on funduscopy and family history checking, we excluded patients with a family history of hypertension and diabetes and finally enrolled 264 patients with DR and 206 patients with non-diabetic retinopathy (NDR). Through correlation analysis, univariate and multivariate regression, we further explore the association between NLR, PLR, and DR. On top of that, we investigate the effect of NLR and PLR on risk reclassification of DR.

**Results:**

Compared with NDR patients, NLR and PLR levels are significantly higher among DR patients (NLR: 2.36 ± 1.16 in DR group versus 1.97 ± 1.06 in NDR group, p < 0.001; PLR: 11.62 ± 4.55 in DR group versus10.56 ± 4.45 in NDR group, p = 0.012). According to univariate analysis, NLR and PLR add risks to DR. After fully adjusting co-founders, NLR, as both continuous and categorical variate, remains an independent risk factor for DR (OR (95%CI): 1.37 (1.06, 1.78) P = 0.018). And though PLR was not independently associated with DR as a continuous variable (OR (95%CI) 1.05 (0.99, 1.11) p = 0.135), the highest quantile of PLR add two-fold increased risk (OR (95%CI) 2.20 (1.05, 4.59) p = 0.037) in the fully adjusted model for DR. In addition, addition of PLR and NLR to the established factor hemoglobin (Hb) improved the discriminability of the model and assisted the reclassification of DR. After combining PLR and NLR the Area under curve (AUC) of Hb based model raised from 0.76 to 0.78, with a category-free net reclassification improvement (NRI) of 0.532 (p < 0.001) and integrated discrimination improvement (IDI) of 0.029 (p < 0.001).

**Conclusions:**

Systemic inflammatory response indexes NLR and PLR were associated with the presence of DR among patients without associated family history and contributed to improvements in reclassification of DR in addition to Hb.

## Background

Diabetic retinopathy (DR), as a specific neurovascular complication of diabetes mellitus(DM),become a major reason for blinding people aged 20–74 [1]. The pathogenesis of DR was complicated, with diverse factors involvement. According to the major researches, commonly acknowledged risk factors include family history, hyperglycemia, hypertension, and hyperlipidemia, long-duration of diabetes, diabetic nephropathy, blood glucose fluctuation, obesity and gestation [2–5]. Particularly, inheritable and unchangeable factors such as genetic polymorphism were closely associated with the incidence and progress of DR [5]. Due to the high costs and low cooperation rate among patients, family history was regarded as a direct or indirect interpretation of inheritable risk factor which helps clinicians to evaluate the risk for DR. It is reported that presence of family history, diabetes, and hypertension, always indicated lower age of the patients which is associated to rather longer duration of diabetes for these patients [6, 7]. In addition, researches revealed a family history of diabetes and hypertension also contributed to a higher frequency of DR [8, 9]. However, in clinical practice, despite the complicated inheritable factors, a large number of diabetic patients without obvious family history also take up a major part of DR patients. Exploring risk factors for these people is quite important for clinical decisions.

Recently, increasing studies reported that routine blood examination could provide rich and effective information to assist risk stratification of disease [10, 11].The promising index neutrophil-to-lymphocyte ratio (NLR) and platelet-to-lymphocyte ratio (PLR) conveyed a systematic inflammatory response in our body and has been evidenced as predictive and prognostic factors for DM and the related complications [12–15]. Moreover, Hemoglobin (Hb) was also a well-established biomarker for DR [16, 17]. However, the association between these factors and DR among patients without diabetes or hypertension family history remains unclear. Herein, our study aimed at clarifying the relationship between NLR, PLR, and DR in patients without inheritable risk factors, and further detect the contribution of NLR and PLR to disease reclassification when combining the traditional factor Hb.

## Methods

### Participants and study design

We consecutively enrolled a total of 1030 type 2 diabetic patients undergoing funduscopy from the endocrinology department of the Second hospital of People in Yun Nan. Diagnosis of type 2 diabetes was made according to the 1999 World Health Organization criteria [18]. The diagnosis of diabetic was based on the 2002 International Clinical Classification Standard [19]. And the non-diabetic retinopathy group (NDR) was defined as patients who had been clearly diagnosed with type 2 diabetes and had no diabetic retinopathy on fundus. Besides patients with a family history of hypertension and diabetes, patients with severe systemic disease, glaucoma, trauma, non-diabetic retinopathy, pregnancy, malignant tumors, severe cardiovascular and cerebrovascular diseases, liver and kidney dysfunction, blood disease, recent surgery, infection or other severe stress condition were also excluded. Finally, we successfully enrolled 264 patients with DR and 206 patients without DR (the NDR group).

### Clinical information and biochemical examination

All participants received routine examination and blood examination and were asked in detail about their disease history, medical history, and personal history. After collecting patients fasting peripheral blood, blood routine test and white blood cell classification were performed. Specifically, the blood routine and blood cell count were detected using a fully automated blood analyzer from Xisen Meikang (XE-2100, Japan); nucleic acid fluorescent staining, laser counting and flow cytometry were used to determine white blood cells and white blood cell classification; colorimetric method was used to determine hemoglobin concentration; and double sheath flow impedance method was applied to determine platelet count and red blood cell count. Neutrophil-to-lymphocyte ratio (NLR), platelet-to-lymphocyte ratio (PLR) and monocyte-to-lymphocyte ratio (MLR) were calculated for all blood samples. All biochemical analyses of the same samples were performed in our hospital, including the analyses of fasting blood glucose (FPG), glycosylated hemoglobin A1c (HbA1c), triglyceride (TG), total cholesterol (TC), low-density lipoprotein cholesterol (LDL-C), high-density lipoprotein cholesterol (HDL-C), serum creatinine (Scr), and blood urea nitrogen (BUN). Among them, the glucose determination kit (glycokinase method) was used to determine fasting blood glucose; the HbA1c determination kit (enzymatic method) was used to determine HbA1c in plasma; the creatinine detection kit (enzymatic method) was used to determine serum creatinine; the urea nitrogen detection kit (Enzymatic method) were used for determination of serum urea nitrogen level. Total cholesterol kit (liquid) (oxidase method), triglyceride kit (liquid) (glycerol phosphate oxidase method), lipoprotein cholesterol detection kit (direct method), and low-density lipoprotein cholesterol detection kit (direct method) were used for measurements of total cholesterol, triglyceride, high-density lipoprotein cholesterol and low-density lipoprotein cholesterol in serum, respectively.

### Statistics analysis

Distribution normality was initially tested through the Kolmogorov–Smirnov test. Continuous data are showed by the mean ± standard deviation (SD) and were compared by independent Student *t* test or one-way analysis of variance test. While variables without normality were expressed by median plus IQR. The Chi squared test or Fisher’s exact test was used to compare categorical variables. Due to the order of magnitude of PLR, a scaling factor of 10 was included to explain unit conversion. The correlation was assessed by the Spearman correlation test. NLR, PLR were analyzed as continuous variates and also respectively divided into quartiles, with the first quartile representing the lowest levels and the fourth quartile the highest. Univariate logistic regression models were used to examine possible factors associated with DR. Multivariable logistic regression analysis was conducted to assess the independent association between NLR, PLR and DR. Variables with known clinical risk, significance at the 5% level from the univariate analyses were included as co-founders in for an adjustment. After adjusting for age, gender, diabetes duration, hypertension, BMI, Scr, white blood cell (WBC), Hb, BUN, TG, FPG and HBA1c, odds ratios with 95% confidence intervals were reported. In logistics regression, an Akaike information criterion (AIC) based stepwise variable selection method was used to acquire the optimal logistic regression model.

All statistical analyses were performed by SPSS software (version 22.0; SPSS, Inc., Chicago, IL, USA). P-values less than 0.05 (P < 0.05) were considered significant and the significance was two-tailed.

## Results

### Baseline

**Fig. 1:**
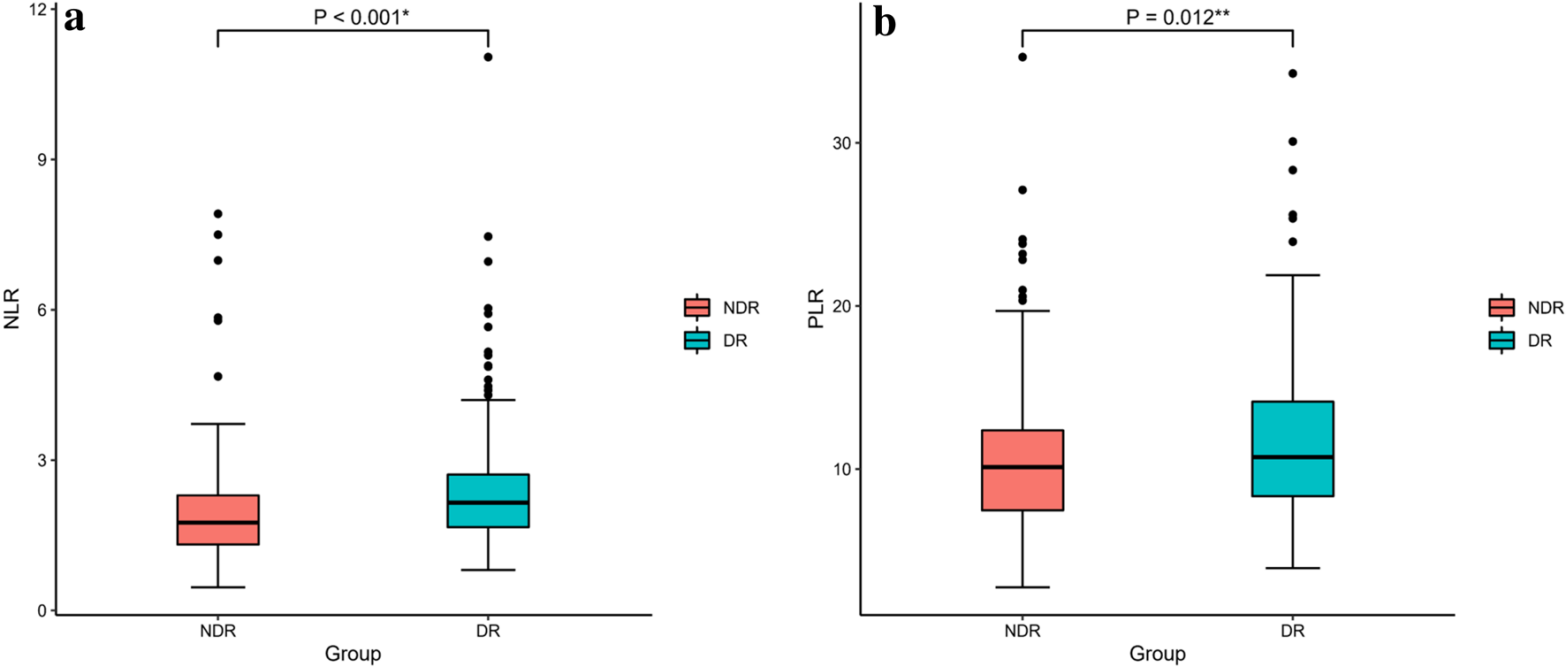
Comparison of NLR and PLR in different group **a** Boxplot showed that DR patients had higher NLR than the NDR patients (p < 0.001**). **b** Boxplot showed that DR patients had higher PLR than the NDR patients (p = 0.012)

As shown in Table 1, gender, presence of hypertension, smoking, drinking, diabetes duration, FPG, WBC, Hb, BUN, Scr and TG are significantly different between DR and NDR group (P < 0.05). Specifically, compared with the NDR group, DR patients have longer diabetes duration, higher levels of FPG, WBC, BUN, Scr and TG, a higher proportion of female and hypertension presence. However, levels of Hb were lower in DR patients. In addition, no difference was observed in age, BMI, HBA1c, TC, LDL-C, HDL-C levels as well as the treatment of diabetes. According to Fig. 1, levels of NLR (2.36 ± 1.16 in DR group versus 1.97 ± 1.06 in NDR group, p < 0.001) and PLR(11.62 ± 4.55 in DR group versus10.56 ± 4.45 in NDR group, p = 0.012)were significantly higher in DR group.

**Table 1:** Baseline characteristics of the DR and NDR groups

Variable	DR group N = 264	NDR group N = 206	*P*
Gender (male/female)	131/133	146/60	0.000**
Hypertension (no/yes)	95/169	108/98	0.000**
Smoking (no/yes)	168/96	107/99	0.007*
Drinking (no/yes)	180/84	119/87	0.013*
Sulfonylureas (no/yes)	147/117	110/96	0.344
Biguanides (no/yes)	129/135	95/110	0.327
Insulin (no/yes)	127/137	108/98	0.201
Age (years)	56.48 ± 9.86	55.44 ± 11.27	0.295
NLR	2.36 ± 1.16	1.97 ± 1.06	0.000**
MLR (*10)	2.28 ± 1.03	2.34 ± 1.28	0.628
PLR (*0.1)	11.62 ± 4.55	10.56 ± 4.45	0.012*
Neutrophils (*10^9^/L)	4.25 ± 1.31	3.72 ± 1.22	0.000**
Lymphocytes (*10^9^/L)	1.98 ± 0.64	2.11 ± 0.67	0.031*
Monocytes (*10^9^/L)	0.42 ± 0.15	0.44 ± 0.15	0.097
Platelets (*10^9^/L)	213.84 ± 69.19	203.90 ± 54.66	0.092
Diabetes course (month)	122.86 ± 87.38	66.16 ± 66.25	0.000**
BMI (kg/m^2^)	24.12 ± 3.25	24.62 ± 3.14	0.090
FPG (mmol/L)	9.60 ± 3.79	8.80 ± 3.27	0.019 *
HbA1c (%)	9.96 ± 2.45	9.68 ± 2.66	0.246
WBC (*10^9^/L)	6.83 ± 1.65	6.45 ± 1.50	0.010*
Hb (g/L)	136.25 ± 19.33	153.20 ± 15.70	0.000**
BUN (mmol/L)	7.07 ± 7.82	5.32 ± 1.64	0.000**
Scr (mmol/L)	84.21 ± 46.63	69.14 ± 17.87	0.000**
TG (mg/dL)	3.02 ± 2.37	2.29 ± 1.99	0.000**
TC (mg/dL)	4.73 ± 2.09	4.79 ± 1.13	0.694
HDL-C (mg/dL)	1.16 ± 0.33	1.12 ± 0.57	0.434
LDL-C (mg/dL)	3.11 ± 0.98	2.97 ± 0.88	0.110

*DR* diabetic retinopathy, *NDR* non-diabetic retinopathy, *NLR* neutrophil-to-lymphocyte ratio, *MLR* monocyte-to-lymphocyte ratio, *PLR* platelet-to-lymphocyte ratio, *BMI* body mass index, *FPG* fasting plasma glucose, *HbA1c* glycated hemoglobin A1c, *WBC* white blood cell, *Hb* hemoglobin, *BUN* blood urea nitrogen,*Scr* serum creatinine,*TG* triglycerides, *TC* total cholesterol, *HDL*-*C* high density lipoprotein cholesterol, *LDL*-*C* low density lipoprotein cholesterol

*P < 0.05, **P < 0.01

### Correlation analysis of NLR, PLR and Hb and major clinical factors

As shown in Table 2, according to correlation analysis, NLR positively correlated with MLR, PLR, diabetes duration, BUN and Scr, and negatively correlated with Hb, but displayed insignificant correlation with age, gender, hypertension, BMI, FPG, and HbA1c. Meanwhile, PLR correlated positively with NLR, MLR and diabetes duration, and negatively with BMI and Hb. In addition, Hb correlated positively with BMI and negatively correlated with gender, hypertension, age, NLR, PLR, diabetes course, and BUN.

**Table 2:** Correlation analysis of NLR, PLR and Hb and major clinical factors

Variable	NLR		PLR (*0.1)		Hb(g/L)	
	R	*P*	R	*P*	R	*P*
Gender (male/female)	−0.061	0.190	0.080	0.082	−0.465	0.000**
Hypertension (no/yes)	0.053	0.247	−0.008	0.855	−0.130	0.005**
Age (years)	0.055	0.238	0.029	0.534	−0.150	0.001**
NLR	1	1	0.520	0.000**	−0.115	0.012*
MLR (*10)	0.609	0.000**	0.446	0.000**	0.004	0.934
PLR (*0.1)	0.520	0.000**	1	1	−0.289	0.000**
Diabetes course (month)	0.125	0.007**	0.093	0.044*	−0.266	0.000**
BMI (kg/m^2^)	−0.021	0.651	−0.117	0.011*	0.194	0.000**
FPG (mmol/L)	0.015	0.755	−0.063	0.184	0.080	0.091
HbA1c (%)	−0.001	0.980	−0.072	0.129	0.076	0.110
Hb (g/L)	−0.115	0.012*	−0.289	0.000**	1	1
BUN (mmol/L)	0.100	0.033*	−0.042	0.371	−0.220	0.000**
Scr (mmol/L)	0.173	0.000**	0.079	0.092	−0.065	0.168
Sulfonylureas (no/yes)	−0.078	0.090	0.049	0.291	-0.016	0.725
Biguanides (no/yes)	0.033	0.476	−0.058	0.209	0.072	0.120
Insulin (no/yes)	0.081	0.081	−0.032	0.491	0.009	0.839

*NLR* neutrophil-to-lymphocyte ratio, *MLR* monocyte-to-lymphocyte ratio, *PLR* platelet-to-lymphocyte ratio, *BMI* body mass index, *FPG* fasting plasma glucose, *HbA1c* glycated hemoglobin A1c, *Hb* hemoglobin, *BUN* blood urea nitrogen, *Scr* serum creatinine, *R* correlation coefficient

*P < 0.05, **P < 0.01

### Univariate analysis

As shown in Table 3, our univariate regression analysis revealed that gender of female, presence of hypertension, long course of diabetes,higher levels of FPG, WBC, NLR, PLR BUN, Scr and TG adds risk to presence of DR, while higher counts of Hb was related to lower risk of DR. However, no association was found between age, BMI, HbA1c, TC, HDL-C, LDL-C, MLR and presence of DR.

**Table 3:** Univariate analysis

Variable	OR	95%CI lower	95%CI upper	*P*
Gender (Male/Female)	2.47	1.68	3.63	0.000**
Hypertension (no/yes)	1.96	1.35	2.84	0.000**
Age (years)	1.01	0.99	1.03	0.295
NLR	1.46	1.19	1.79	0.000**
MLR (*10)	0.96	0.82	1.13	0.619
PLR (*0.1)	1.06	1.01	1.10	0.013*
Diabetes course (month)	1.01	1.01	1.01	0.000**
BMI (Kg/m2)	0.95	0.90	1.01	0.090
FPG (mmol/L)	1.07	1.01	1.13	0.020*
HbA1c (%)	1.05	0.97	1.13	0.239
WBC (*10^9^/L)	1.17	1.04	1.31	0.010*
Hb (g/L)	0.95	0.93	0.96	0.000**
BUN (mmol/L)	1.42	1.26	1.59	0.000**
Scr (mmol/L)	1.02	1.01	1.02	0.000**
TG (mg/dL)	1.18	1.07	1.31	0.001**
TC (mg/dL)	0.98	0.88	1.09	0.693
HDL-C (mg/dL)	1.21	0.76	1.93	0.412
LDL-C (mg/dL)	1.17	0.12	1.43	0.110

*NLR* neutrophil-to-lymphocyte ratio, *MLR* monocyte-to-lymphocyte ratio, *PLR* platelet-to-lymphocyte ratio, *BMI* body mass index, *FPG* fasting plasma glucose, *HbA1c* glycated hemoglobin A1c, *WBC* white blood cell, *Hb* Hemoglobin, *BUN* blood urea nitrogen,*Scr* serum creatinine,*TG* triglycerides, *TC* total cholesterol, *HDL*-*C* high density lipoprotein cholesterol, *LDL*-*C* low density lipoprotein cholesterol, *OR* odds ratios, *CI* confidence intervals

*P < 0.05, **P < 0.01

### Associations between NLR, PLR, and DR

#### NLR and DR

By conducting multivariate analysis, we found that NLR was associated with DR independent of other known factors. With a unit increase of NLR, the risk for DR would raise 37%. Furthermore, when treated as a category variate divided according to its quantile, the association of NLR and DR still exists. As Table 4 demonstrated, from the crude model to simple or complex model, there was a 2.8 fold increased risk for DR in the highest quantile of NLR (OR, 95%CI 2.80 (1.32, 5.95) p = 0.007) (Table 4).

**Table 4:** Independent association between NLR and DR

Exposure	Model	Model I	Model II
NLR group	OR (95%CI) *P*	OR (95%CI) *P*	OR (95%CI) *P*
ContinuousNLR	1.46 (1.19,1.78) 0.000**	1.46 (1.18,1.81) 0.000**	1.37 (1.06,1.78) 0.018*
0.46–1.49	1	1	1
1.50-1.95	1.89 (1.12,3.17) 0.016*	2.11 (1.19,3.73) 0.011*	1.23 (0.62, 2.44) 0.556
1.95–2.54	2.25 (1.33,3.79) 0.002**	2.51 (1.42,4.46) 0.002**	1.77 (0.88, 3.55) 0.110
2.54–11.05	3.44 (2.01,5.89) 0.000**	3.61 (1.99,6.55) 0.000**	2.80 (1.32, 5.95) 0.007**
P for trend	0.000**	0.000**	0.004**

Model: crude model; Model I: adjusting for age, gender, diabetes duration; Model II: adjusting for age, gender, diabetes duration, hypertension, BMI, Scr, WBC, Hb, BUN, TG, FPG and HbA1c

*NLR* neutrophil-to-lymphocyte ratio,*OR* odds ratios, *CI* confidence intervals, *BMI* body mass index, *Scr* serum creatinine, *WBC* white blood cell, *Hb* hemoglobin, *BUN* blood urea nitrogen, *TG* triglycerides, *FPG* fasting plasma glucose, *HbA1c* glycated hemoglobin A1c

*P < 0.05, **P < 0.01

#### PLR and DR

We also included PLR in multivariate logistics regression by adjusting other co-founders and observed that PLR was not an independent risk factor for DR as a continuous variable, but the ranked level of the index assists the risk stratification. Specifically, after full adjustment, the highest quantile of PLR held add 2.2 times of risk to the presence of DR compared with the first quantile(OR(95%CI)2.20 (1.05, 4.59), P = 0.037) (Table 5).

**Table 5:** Independent association between PLR and DR

Exposure	Model	Model I	Model II
PLRgroup	OR (95%CI) *P*	OR (95%CI) *P*	OR (95%CI)
Continuous PLR	1.06 (1.01,1.10) 0.013*	1.04 (0.99,1.09) 0.109	1.05 (0.99, 1.11) 0.135
2.75–7.98	1	1	1
7.99–10.34	1.65 (0.98, 2.76) 0.058	1.86 (1.06, 3.27) 0.031*	1.37 (0.70, 2.71) 0.360
10.36–13.40	1.17 (0.70, 1.94) 0.558	1.34 (0.77, 2.34) 0.307	0.95 (0.47, 1.92) 0.891
13.49–35.26	2.51 (1.47, 4.27) 0.000**	2.15 (1.20, 3.85) 0.001**	2.20 (1.05, 4.59) 0.037*
P for trend	0.003**	0.033*	0.073

Model: crude model; Model I: adjusting for age, gender, diabetes duration; Model II: adjusting for age, gender, diabetes duration, hypertension, BMI, Scr, WBC, Hb, BUN, TG, FPG and HbA1c

*PLR* platelet-to-lymphocyte ratio, *OR* odds ratios, *CI* confidence intervals, *BMI* body mass index, *Scr* serum creatinine, *WBC* white blood cell, *Hb* hemoglobin, *BUN* blood urea nitrogen, *TG* triglycerides, *FPG* fasting plasma glucose, *HbA1c* glycated hemoglobin A1c

*P < 0.05, **P < 0.01

### NLR and PLR for reclassification of DR

#### Hb and DR

Particularly, we also assess the performance of the previous factor-Hb in our diabetic patients’ group. By applying crude and different multivariate models, we confirmed that no matter treated as a continuous variable or categorical variable, Hb was stably related to lower risk of DR with the higher quartile displaying lower risk probability (Table 6).

**Table 6:** Independent association between Hb and DR

Exposure	Model	Model I	Model II
Hb group	OR (95%CI) *P*	OR (95%CI) *P*	OR (95%CI) *P*
Continuous Hb	0.95 (0.93, 0.96) 0.000**	0.95 (0.94,0.97) 0.000**	0.96 (0.94, 0.97) 0.000**
70–132(g/L)	1	1	1
133–143(g/L)	0.32 (0.17, 0.61) 0.000**	0.38 (0.19, 0.73) 0.004**	0.88 (0.44, 1.74) 0.707
144–157(g/L)	1.61 (0.95, 2.71) 0.076	2.02 (1.12, 3.62) 0.019*	0.27 (0.12, 0.60) 0.001**
158–195(g/L)	0.07 (0.04, 0.13) 0.000**	0.09 (0.05,0.20) 0.000**	0.95 (0.93, 0.97) 0.000**
P for trend	0.000**	0.000**	0.000**

Model: Univariate model; Model I: adjusting for age, gender, and diabetes course; Model II: adjusting for age, gender, diabetes course, hypertension, BMI, Scr, WBC, NLR, PLR, BUN, TG, FPG, HbA1C

*Hb* hemoglobin, *OR* odds ratios, *CI* confidence intervals, *BMI* body mass index, *Scr* serum creatinine, *WBC* white blood cell, *NLR* neutrophil-to-lymphocyte ratio, *PLR* platelet-to-lymphocyte ratio, *BUN* blood urea nitrogen, *TG* triglycerides, *FPG* fasting plasma glucose, *HbA1c* glycated hemoglobin A1c

*P < 0.05, **P < 0.01

### A combination of PLR, NLR, and Hb for predicting DR

**Fig. 2:**
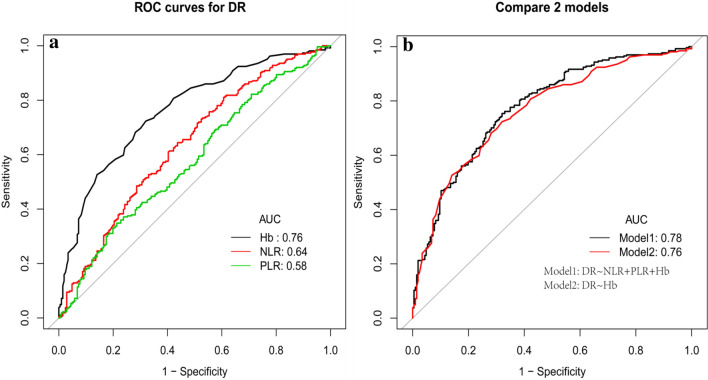
Receiver operating characteristic (ROC) curves of NLR, PLR and Hb for DR **a** The ROC curves using Hb, NLR or PLR alone for discriminating DR (Hb, black, NLR, red, PLR, green) **b** The ROC curves included and not included NLR and PLR for predicting DR, (Model1: black, a combination of NLR, PLR and Hb for predicting DR, Model2: red, using Hb along to predict DR)

In order to evaluate the prognostic value of NLR and PLR for improving risk stratification of DR, we performed a receiver-operating characteristic (ROC) analyses to calculate the area under the curve (AUC) of each factor and assess the performance of a combination of these factors (Table 7). Though NLR and PLR alone didn’t perform better than Hb, a combination of NLR, PLR, and Hb indeed result in a model with increased predictive performance (area under the ROC curve 0.78 (95%CI 0.74–0.82) versus. 0.76 (95%CI 0.72–0.81)) (Fig. 2). Furthermore, the addition of NLR, PLR significantly improved the risk reclassification over using Hb alone, with a considerable category-free net reclassification improvement(NRI) and a meaningful integrated discrimination improvement (IDI) for DR among diabetic patients without a family history.(NRI(95%CI) 0.53 (0.36–0.71) p < 0.001; IDI(95%CI) 0.03 (0.01–0.04); p < 0.001.)

**Table 7:** A combination of PLR, NLR and Hb for predicting DR

Marker	AUC	95%CI lower	95%CI upper	Cut off	Specificity	Sensitivity
NLR	0.64	0.58	0.69	1.84	0.56	0.64
PLR	0.58	0.53	0.63	128.11	0.79	0.35
Hb	0.76	0.72	0.81	146.50	0.68	0.72
NLR + PLR + Hb	0.78	0.74	0.82	–	0.68	0.75

Based on model: logit (DR) = 8.74874−0.06060*Hb +0.49400*NLR−0.00682*PLR

*AUC* area under curve*, CI* confidence intervals, *NLR* neutrophil-to-lymphocyte ratio, *PLR* platelet-to-lymphocyte ratio, *Hb* hemoglobin, *NRI* net reclassification index, *IDI* integrated discrimination improvement

## Discussion

Our research demonstrated the association of systemic inflammatory response index with diabetic retinopathy among type 2 diabetic patients without related family histories. First of all, we verified that levels of NLR and PLR but not MLR were higher in the DR group. Furthermore, according to our multivariate analysis, not only did NRL serve as an independent risk factor but also the highest quartile of both NLR and PLR added risk to DR. More importantly, addition of NLR and PLR to Hb-based model improved reclassification of DR. Through our study, we provide the simple and available blood-based index for DR, promoting the risk stratification of DR among type 2 diabetic patients without family history.

Although the association between blood inflammatory index and DR drew much attention previously [12, 20–22],our study further deciphered the association and the clinical application of these indicators in T2DM patients without associated family history. Herein, we investigate the association between the indicators and DR more comprehensively in a larger population (n = 470) and revealed that a combination of NLR, PLR and Hb displayed significantly improved discriminability and raised sensitivity compared with using Hb alone. Therefore, combining the three factors might be helpful in clinical practice to improve the identification of DR in T2DM patients without family history of diabetes and hypertension.

Chronic inflammation plays an essential role in the initiation and progression of type 2 diabetes and further accelerates the deterioration of micro-angiopathy and macrovascular disease in patients with diabetes [23]. Previous studies have evidenced that peripheral blood leukocytes and their subgroups are associated with macrovascular and microvascular complications among patients with type 2 diabetes [24]. Specifically, peripheral blood leukocytes include lymphocytes, basophils, neutrophils, eosinophils, and monocytes, each type of which holds a unique biological function in systemic inflammation. NLR and PLR are two indexes that represent the integration of two factors and are considered to be new markers of the systemic inflammatory response [14]. Increasing studies have confirmed their association with type 2 diabetes [14, 25]. DR is a common micro-angiopathic complication in diabetes. More and more evidence indicates that inflammation plays an important part in the early and progressive stage of DR [26–28], through inducing the formation of new blood vessels and macular edema [26], damaging the glial crosstalk and causing neuronal loss [29]. In addition, studies have also found that many inflammatory cytokines (such as CRP, TNF-α and VEGF, etc.) increase in patients with DR [30]. And intervention and regulation targeted at the inflammatory response in patients with diabetic retinopathy [31] can prohibit the progression of diabetes and retinopathy.

As two major indexes related to systemic inflammatory, PLR and NLR have been proved to be associated with diabetes and its complications [12, 14]. Apart from genetic epigenetic factors (family history of diabetes and hypertension), our study showed that higher NLR levels significantly increased the risk of DR, which is consistent with the previous results. But compared with the former one, our research has a larger sample size with an enrollment of 470 patients and also excludes the inheritable family history. NLR represents peripheral blood neutrophils to lymphocytes ratio, which integrates different but complementary immune pathways in circulating blood. First of all, elevated NLR can be a manifestation of an increased number of neutrophils, which adhere to the endothelial cell, leading to vascular endothelial damage, and in turn causing extensive chronic inflammation [11, 32]. Hence the NLR might reveal the enhanced microvascular inflammation in DR patients. Secondly, lymphocytes serve as a major part of the body’s immune response. They have the ability to control and regulate inflammatory responses, and a relatively higher proportion of CD4 T cells were proved to be anti-atherosclerotic [33]. And our study found a decrease in absolute count of lymphocytes among peripheral blood in patients with high-level NLR (NLR:2.36 ± 1.16 in DR group versus 1.97 ± 1.06 in NDR group, p < 0.001;absolute count of lymphocytes:(1.98 ± 0.64)*10^9^/L in DR group versus (2.11 ± 0.67)*10^9^/L in NDR group, p = 0.031), which indicates the possibility of insufficient immunoregulation due to the fewer lymphocytes, In addition, this study also demonstrated that NLR levels were positively correlated with the duration of diabetes, BUN, and Scr, which is consistent with the previous results [34].

Previous studies have found that PLR is closely related to diabetes and can be used to assess the progress of the disease [35], predict and evaluate the diabetes-related lower limb vascular disease [15], atherosclerosis and diabetic foot ulcers [36]. However, Atak et al. didn’t found an association between PLR and DR [35], which might be attributed to the small sample size. In our study, though the PLR as a continuous variate didn’t show independent association with DR, the highest quartile of PLR indeed adds more than 2-fold risk to the presence of DR. It has been commonly admitted that platelets participate in thrombosis. Moreover, until now, increasing studies have proved that platelets played an important role in the immuno-inflammatory response. Specifically, platelets can release a variety of immune-regulating cytokines, chemokines, and other mediators, thus regulating the inflammation response in blood vessels in an autocrine or paracrine manner [37]. Meanwhile, it could also regulate neutrophils, endothelial cells, and lymph directly, allowing them to recruit towards injured tissue [38]. Similarly, based on the regulatory function of platelets, increased PLR might represent the relatively active inflammatory response of platelets among DR patients. Additionally, our study also indicates a positive correlation between PLR and diabetes courses and a negative correlation between PLR and BMI. Taken together, we provide the large-sample size-based evidence for the role of PLR in DR, and further mechanism studies are also needed in the future.

We also proved that in diabetic patients without a family history of diabetes and hypertension, Hb levels were still negatively associated with the risk of DR independent of established factors. Hemoglobin is a special protein transporting oxygen within red blood cells. And low hemoglobin levels might lead to tissue ischemia and hypoxia, which is one of the key mechanisms of DR occurrence [39]. Studies have found that hemoglobin levels were negatively related to endothelial function and lower hemoglobin levels directly resulted in organ damage [40]. In addition, the level of hemoglobin is an indicator of the anemia condition in our body. According to previous studies, it has been found that the anemia patients held a high level of vascular endothelial growth factor (VEGF) which is closely related to retinal neovascularization [41, 42]. Some studies indicated that anemia may enhance oxidative stress [43], because the antioxidant capacity of red cells can be damaged due to anemia [44], which in turn promotes oxidative stress and accelerates presence of DR [45]. In particular, in this study, a combination of systemic inflammation indicators NLR, PLR, and Hb was shown to increase the predictability of DR and help DR reclassification.

The established relationships between some clinical factors and DR in our patient’s group were in line with previous ones. Firstly, we found that blood lipid was a risk factor for DR, and the results of this study are consistent with previous one [46], indicating higher TG increased the risk of DR. In addition, in consistency with previous studies, diabetic nephropathy significantly increases the risk of DR with shared pathological mechanism [47, 48]. We also observed higher levels of BUN and Scr in DR patients, revealing a potential interaction multi-organ complication under the background of diabetes.

In summary, in people with type 2 diabetes without a family history of diabetes and hypertension, systemic inflammation indicators NLR and PLR are closely related to DR. Higher NLR and PLR increase the risk of DR, and after combined with Hb indicators, they contributed to DR reclassification. To provide more practical and reliable guidance for clinical diagnosis, further multi-center prospective clinical studies and basic researches are also required to elucidate the relationship between the PLR NLR and DR.

## Limitations

A series of limitations still exist in our study. First of all, our study is a single-center study, patients’ recruitment, staff characteristics, and departmental protocols might add a limitation to the universality of our results. Additionally, with a lack of follow-up, we were unable to verify the predictive value of the factors, the further prospective cohort is still required to clarify the value PLR, NLR in predicting DR among T2DM patients in clinical practice. Finally, as our participants were type 2 diabetic patients without family history of hyperextension and diabetes, the results might not be extrapolated to other populations.

## Conclusion

Systemic inflammatory response indexes NLR and PLR were associated with the presence of DR among patients without related family history and improved discriminability and re-classification of hemoglobin-based predictive model.

## Data Availability

The datasets used during the present study are available from the corresponding author on reasonable request.

## Abbreviations

DR: Diabetic retinopathy
DM: Diabetes mellitus
NLR: Neutrophil-to-lymphocyte ratio
PLR: Platelet-to-lymphocyte ratio
T2DM: Type 2 diabetes mellitus
NDR: Non-diabetic retinopathy
Hb: Hemoglobin
AUC: Area under curve
NRI: Net reclassification improvement
IDI: Integrated discrimination improvement
MLR: Monocyte-to-lymphocyte ratio
FPG: Fasting blood glucose
HbA1c: Glycosylated hemoglobin A1c
TG: Triglyceride
TC: Total cholesterol
LDL-C: Low-density lipoprotein cholesterol
HDL-C: High-density lipoprotein cholesterol
Scr: Serum creatinine
BUN: Blood urea nitrogen
SD: Standard deviation
WBC: White blood cell
AIC: Akaike information criterion
BMI: Body mass index
